# Transplantation of Human Cortically-Specified Neuroepithelial Progenitor Cells Leads to Improved Functional Outcomes in a Mouse Model of Stroke

**DOI:** 10.3389/fncel.2021.654290

**Published:** 2021-04-29

**Authors:** Rehnuma Islam, Stasja Drecun, Balazs V. Varga, Ilan Vonderwalde, Ricky Siu, Andras Nagy, Cindi M. Morshead

**Affiliations:** ^1^Faculty of Medicine, Institute of Medical Science, University of Toronto, Toronto, ON, Canada; ^2^Institute of Biomedical Engineering, University of Toronto, Toronto, ON, Canada; ^3^Wellcome Trust-Medical Research Council Cambridge Stem Cell Institute, University of Cambridge, Cambridge, United Kingdom; ^4^Department of Surgery, University of Toronto, Toronto, ON, Canada; ^5^Lunenfeld-Tanenbaum Research Institute, Mount Sinai Hospital, Toronto, ON, Canada

**Keywords:** stroke, mouse behavior, human stem cell transplantation, stem cell survival and differentiation, human neuroepithelial cell, immunogenic response

## Abstract

Stroke is a leading cause of death and long-term disability worldwide. Current therapeutic options are limited in terms of their time for implementation and efficacy in promoting recovery. Cell transplantation has been shown to have promise in several animal models however significant challenges remain, including the optimal source of cells to promote neural repair. Here, we report on the use of a population of human ESC derived, cortically specified, neuroepithelial precursor cells (cNEPs) that are neurally restricted in their lineage potential. CNEPs have the potential to give rise to mature neural cell types following transplantation, including neurons, astrocytes and oligodendrocytes. With a view towards translation, we sought to determine whether this human cell source was effective in promoting improved functional outcomes following stroke. Undifferentiated cNEPs were transplanted in a pre-clinical endothelin-1 (ET-1) model of ischemic motor cortical stroke in immunocompromised SCID-beige mice and cellular and functional outcomes were assessed. We demonstrate that cNEP transplantation in the acute phase (4 days post-stroke) improves motor function as early as 20 days post-stroke, compared to stroke-injured, non-transplanted mice. At the time of recovery, a small fraction (<6%) of the transplanted cNEPs are observed within the stroke injury site. The surviving cells expressed the immature neuronal marker, doublecortin, with no differentiation into mature neural phenotypes. At longer survival times (40 days), the majority of recovered, transplanted mice had a complete absence of surviving cNEPS. Hence, human cNEPs grafted at early times post-stroke support the observed functional recovery following ET-1 stroke but their persistence is not required, thereby supporting a by-stander effect rather than cell replacement.

## Introduction

Stroke is a leading cause of death and disability in adults (Khoshnam et al., 2017). Current treatment options are limited and do not promote complete recovery after stroke, leaving patients with poor quality of life and socioeconomic hardship. One potential treatment to improve outcome following stroke involves transplantation of stem and progenitor cells (termed precursor cells). This approach has been shown to be effective in animal models of stroke (Mohamad et al., 2013; Xiong et al., 2017; Payne et al., 2018; Vonderwalde et al., 2019), however, several hurdles remain, including the identification of an optimal cell source to promote neural repair and functional recovery.

A variety of cell sources have been explored in transplant studies to treat stroke. Embryonic stem cells (ESCs) and induced pluripotent stem cells (iPSCs) can give rise to all cell types in the central nervous system (CNS), however they have reported tumorigenic capacity upon transplantation, limiting their efficacy (Thomson et al., 1998; Gao et al., 2016a). A recent approach to reducing the tumorigenic potential of transplanted cells involves the use of genome-editing strategies to insert a suicide gene into a cell division essential locus, allowing selective ablation of proliferating cells through administration of a pro-drug (Payne et al., 2019a). Other approaches to circumvent tumorigenicity include the transplantation of neurally committed cells with less tumorigenic potential (Gao et al., 2016b). For stroke application, previous studies have shown efficacy of both mesenchymal stem cells and neural precursors in improving motor recovery by enhancing host neuroplasticity as measured by increased synapse formation, without the need for integration and maturation (Wakabayashi et al., 2009; Oki et al., 2012; Vonderwalde et al., 2019). Additionally, studies suggest that neural stem cells mediate recovery through maturation and integration of transplanted cells (Zhou et al., 2015; Ficek et al., 2016). Herein, we have examined the potential of human ESC derived, cortical neuroepithelial cells (cNEPs). These neurally committed cells are present during brain development and have the capacity to generate cortical neural cell types *in vitro*. Hence, cNEPs provide regionally specific brain cells for transplantation in a cortical stroke injury model (Tornero et al., 2017; Payne et al., 2019b) and the opportunity to evaluate whether cNEPs promote improved functional outcomes by examining grafted cell survival, maturation and integration.

CNEPs are found in the developing neural tube, where they generate radial glia progenitors, neurons, astrocytes and oligodendrocytes (Martínez-Cerdeño and Noctor, 2018). *In vitro*, cNEPS can be derived from pluripotent stem cells, including iPSCs and ESCs (Guillaume et al., 2006; Payne et al., 2018). A previous study used iPSC derived cNEPS in a rat model of motor stroke that lesioned the cortex and striatum, and demonstrated minimal improvements in functional outcomes when transplanted in a hydrogel (Payne et al., 2019b). Whether ESC derived cNEPs can improve stroke outcomes in a mouse model of motor cortex stroke, has not been explored. Further, with a view towards translation and in accordance with stroke therapy academic industry roundtable (STAIRS) and the stem cell therapies as an emerging paradigm in stroke (STEPS) guidelines, it is important to demonstrate the efficacy of cNEPs in more than one model of stroke to advance therapeutics (Lapchak et al., 2013).

Here, we use an endothelin-1 (ET-1) model of focal ischemic stroke in the motor cortex that mimics the human condition. ET-1 is a potent vasoconstrictor that results in the formation of a lesion cavity, surrounding penumbra and formation of a glial scar—hallmarks of the cellular response to ischemic insult (Adkins et al., 2004; Horie et al., 2008; Erlandsson et al., 2011; Sachewsky et al., 2014). The model is reproducible and generates measurable functional impairments that permit the evaluation of the efficacy of transplanted cells in promoting neural repair. CNEPs were transplanted on post-stroke day 4 (PSD4; acute phase) and we observed a rapid and significant motor recovery by PSD20. Interestingly, at the time of recovery, the vast majority of cNEPS were no longer present in the injured tissue and surviving transplanted cells had immature neural phenotypes. Groups of mice that survived to PSD40 continued to display functional recovery in the absence of cell maturation and further, 67% of the recovered mice were devoid of surviving transplanted cells. Our study demonstrates that integration and maturation of cNEPs is not necessary for the recovery observed in this ET-1 model of stroke.

## Materials and Methods

### H1 ESC Induction

Human H1 ESC (WiCell, Madison, WI, USA) were grown on Geltrex-coated (Thermo Fisher Scientific, Waltham, MA, USA) culture plates in mTESR1 medium (StemCell Technologies, Vancouver, BC, Canada) containing 1% PenStrep (Sigma–Aldrich, St. Louis, MO, USA). Cells were lifted with TrypLE (Thermo Fisher Scientific, Waltham, MA, USA) and passaged at 1.5 × 10^4^ cells per cm^2^, into induction and maintenance media containing a cocktail of small molecule inhibitors to generate cNEPs, as previously described (Payne et al., 2018; Varga et al., 2021). Briefly, H1 ESC were induced into cNEPs using media containing 50% DMEM-F12, 50% Neurobasal (Thermo Fisher Scientific), 0.5× N2 supplement (Gibco), 0.5× B27 supplement (Gibco), 1 mM Glutamax (Thermo Fisher Scientific), 25 mM 2-mercaptoethanol (Sigma). H1 ESC were passaged in media supplemented with 10 μM SB431542 and 100 nM LDN193189 and 10 μM Y27632. Media was changed every other day and kept for 8 days prior to passaging into maintenance media.

### CNEP *In vitro* Culture and Differentiation

CNEPs were cultured on laminin-coated culture plates, lifted with TrypLE (Thermo Fisher Scientific, Waltham MA, USA) and passaged at 3 × 10^4^ cells/cm^2^ for 4–10 passages prior to transplantation. For transplant, cNEPs were pelleted and resuspended in regular artificial cerebrospinal fluid (aCSF) at 1 × 10^5^ cells/μl and kept on ice for up to 2 h before intracranial injection. Media contained 50% DMEM-F12, 50% Neurobasal, 25 mM 2-mercaptoethanol, 1 mM Glutamax, 1× N2 supplement, 0.05× B27 minus vitamin A supplement (Gibco), further supplemented with 2 μM CHIR99021 (Peprotech), 1 μM XAV939, 1 μM SB431542 (Peprotech), 10 ng/ml FGF2 (PeproTech), 50 nM LDN193189, 50 nM K02288, 50 nM AKTiVIII (Calbiochem) and 75 nM MK2206 (all other material from Selleck Chem). For transplant, cNEPs were pelleted and resuspended in regular artificial cerebrospinal fluid (aCSF) at 1 × 10^5^ cells/μl and kept on ice for up to 2 h before intracranial injection.

For *in vitro* characterization, cNEPs were cultured in differentiation media containing 50% DMEM/F12 (Thermo Fisher Scientific, Waltham, MA, USA), 50% Neurobasal (Thermo Fisher Scientific, Waltham, MA, USA), 0.2× Insulin with Zinc (Thermo Fisher Scientific, Waltham, MA, USA), 0.2× N2 supplement (National Library of Medicine (2017), 0.1× B27 with Vitamin A (Thermo Fisher Scientific, Waltham, MA, USA), 25 mM 2-Mercaptoethanol (Sigma–Aldrich, St. Louis, MO, USA), 1 mM Glutamax (Thermo Fisher Scientific, Waltham, MA, USA) and 0.075× FBS (Wisent, Saint-Jean-Baptiste, QC, Canada). Media was changed every other day for up to 14 days. Cells were cultured in maintenance medium and fixed 48 h after plating (undifferentiated cells) or 14 days after plating (differentiated cells) with 4% paraformaldehyde, followed by immunocytochemistry. Cells were blocked with 5% normal goal serum (Sigma–Aldrich, St. Louis, MO, USA) in PBS and 0.5% TritonX-100 (Sigma–Aldrich, St. Louis, MO, USA) for 1 h at room temperature. Cells were incubated with primary antibodies diluted in blocking solution, overnight at 4°C. Immunocytochemistry was performed using DAPI (Invitrogen, Carlsbad, CA, USA), anti-OCT4 (1:200; BD Biosciences, 611202), anti-human Nestin (1:50; Millipore, Sigma, ABD69), anti-SOX2 (1:1,000; Abcam, AB97959), anti-BIII tubulin (1:1,000; Sigma, T8860), anti-DCX (1:250; AB18723), anti-Olig2 (1:200; AB9610) and anti-GFAP (1:1,000; Dako, Z0334). Secondary antibodies, Alexa Fluor488 (Invitrogen, Carlsbad, CA, USA) and Alexa Fluor568 (Invitrogen, Carlsbad, CA, USA) were diluted in 5% NGS. Cell markers colocalized with DAPI were counted from three images per well for differentiated cNEP cell counts.

### Animals

Male Fox Chase SCID/Beige (Jackson Labs, Bar Harbor, ME, USA) mice, aged 10–15 weeks were used for all studies. Mice were housed with *ad libitum* food and water. Following stroke surgery, mice were housed individually. All experiments were conducted in accordance with the University of Toronto, Temerty Faculty of Medicine Animal Care Committee and with Canadian Council on Animal Care guidelines.

### ET-1 Stroke

ET-1 stroke was performed as previously described (Vonderwalde et al., 2019). Briefly, the skull was exposed, a small burr hole was drilled at the site of the right sensorimotor cortex at AP: + 0.6 mm, ML: −2.2 mm lateral to bregma and DV: −1.0 mm. Mice received a 1 μl injection of 800 picomolar ET-1 in distilled H_2_O (Millipore, Sigma, St. Louis, MO, USA) using a 2.5 μl Hamilton Syringe with a 26 gauge, 0.375′′ long needle (Hamilton, Reno, NV, USA). ET-1 was injected at a rate of 0.1 μl/min. The needle was removed 10 min after the last injection. Mice were given saline, 5 mg/kg meloxicam as analgesic and allowed to recover. Mice were divided into two groups, Stroke alone and Stroke + cNEP transplant.

### CNEP Transplant

On PSD4, mice in the Stroke + cNEP group received cNEP transplants. CNEPs were lifted with TrypLE (Thermo Fisher Scientific, Waltham, MA, USA) and added 1:1 with DMEM containing 10% FBS (Wisent, Saint-Jean-Baptiste, QC, Canada), counted, pelleted, and resuspended in aCSF at a concentration of 1 × 10^5^ cells/μl, as previously described (Payne et al., 2019b). Cells were injected through the previously drilled burr hole at the same coordinates as the ET-1. One-hundred thousand cells in 1 μl of a CSF was injected using a Hamilton syringe. Control mice received 1 μl of a CSF.

### Tissue Processing and Analysis

On PSD4, PSD8, PSD20 and PSD40, mice were deeply anaesthetized with 250 mg/kg Avertin (Sigma–Aldrich, St. Louis, MO, USA) and intracardially perfused with phosphate buffered saline (PBS) (10 ml/min for 3 min), followed by 4% paraformaldehyde at a rate of 6 ml/min for 5 min. Brains were removed and post-fixed at 4°C overnight in 4% paraformaldehyde and then stored in 30% sucrose until use. Brains were cryosectioned (−20°C) at 20 μm sections, placed on SuperFrost slides (Thermo Fisher Scientific, Waltham, MA, USA) and stored at −20°C.

At the time of processing, sections were washed with PBS and blocked using 1% bovine serum albumin (Sigma–Aldrich, St. Louis, MO, USA), 4% normal goal serum (Sigma–Aldrich, St. Louis, MO, USA) and 0.5% TritonX-100 (Sigma–Aldrich, St. Louis, MO, USA) in PBS for 1 h at room temperature. Sections were incubated with primary antibodies: anti-human Nestin (1:50; Millipore, Sigma, ABD69), anti-HuNu (1:200; Millipore, Sigma, MAB1281), anti-SOX2 (1:1,000; Abcam, AB97959), anti-DCX (1:250; AB18723), anti-NeuN (1:100; Millipore, Sigma, ABN78), anti-GFAP (1:400, Sigma, G3893), anti-MBP (1:50; Abcam, AB7349), anti-Ki67 (1:200, AB15580) and anti-Iba1 (1:500, Wako 019–19741) in blocking solution overnight at 4°C. After 3 × 5 min PBS washes, tissue was incubated with secondary antibodies, Alexafluor488 and 568 (1:1,000; Invitrogen) at room temperature for 1 h. Another 3× PBS washes were performed and sections were mounted using Vectashield with DAPI (Vector, H-1,200). Images were acquired on an inverted Zeiss LSM880 laser scanning confocal and on an Axio Observer Zeiss microscope at 10× and 20× magnification for counting. Colocalized antibody staining with DAPI was counted manually. All brain sections through the stroke injury site were assessed and all sections (range 1–10 sections/brain) that contained transplanted cells were quantified. Cell counts were performed on sections from both treatment groups surrounding the stroke lesion and from sections containing HuNu+ cells from transplanted mice. Three 400 μm × 400 μm areas surrounding the stroke lesion (lateral, medial and inferior) were counted. For Iba1+ cells, the total numbers, as well as those ameboid in shape (with one or less processes), were counted in the same three areas surrounding the lesion and in a minimum of three sections per brain from both treatment groups.

### Histology

The sections were hydrated for 5 min each in 100% ethanol, 95% ethanol and 70% ethanol. Then, rinsed in distilled H_2_O for 1 min and stained using 0.25% cresyl violet in 0.0025% glacial acetic acid and distilled H_2_O for 15 min. Wash steps involved 2 min each of submersion in distilled H_2_O and 95% ethanol. The sections were then placed in 0.25% glacial acetic acid in 95% ethanol for 1 min, dehydrated for 20 s in 100% ethanol, cleared using xylene and mounted using DPX mounting medium (Sigma–Aldrich, St. Louis, MO, USA). Images were taken at 5× magnification on an Axio Observer Zeiss microscope. AxioVision software and ImageJ were used to trace the stroke lesion as previously described (Vonderwalde et al., 2019) and volume was calculated using the average lesion area × the number of sections with lesions × the distance between the sections (160 μm).

### Foot-Fault

The foot fault test was performed to assess motor function and coordination. Mice were placed on a metal grid (1 cm spaces) suspended 12 inches above a table surface and video recorded while mice traversed the grid for 3 min. Uninjured animals demonstrate good coordination and slip minimally during this task. The number of steps and the number of foot slips made with the forelimbs were counted and the difference in foot slip ratio was calculated as (number of contralateral paw slips − number of ipsilateral paw slips)/total number of steps × 100. Mice that did not exhibit a behavioral deficit in foot-fault greater than 1 standard deviation from their mean baseline values were removed from further analysis.

### Catwalk

Catwalk was performed by placing mice at the entrance of a narrow glass walkway with a dark goal box on the opposite end of the walkway. As mice walked across the glass walkway to the goal box, paw prints as beam breaks were recorded on a video camera placed below the walkway. CatwalkXT software v10.6 (Noldus) was used to analyze print data and obtain quantitative parameters for gait analysis (Kappos et al., 2017). Terminal dual stance measured the duration of simultaneous contact of contralateral paws. Support Lateral measured the percent of the walk where simultaneous contact was made with lateral hindlimb and forelimb.

### Statistical Analysis

Statistical analysis was conducted using V6 GraphPAD Prism and IBM SPSS v23. Foot fault and Catwalk were analyzed by repeated measures ANOVA, Tukey’s *post hoc* test and across group comparisons at individual timepoints were analyzed with Student’s *T*-test, Mann–Whitney *post hoc*. All data was represented as mean ± SEM. Significant differences are considered *p* < 0.05.

## Results

### CNEPs Are Multipotent, Neurally Committed Cells *In vitro*

**Figure 1:**
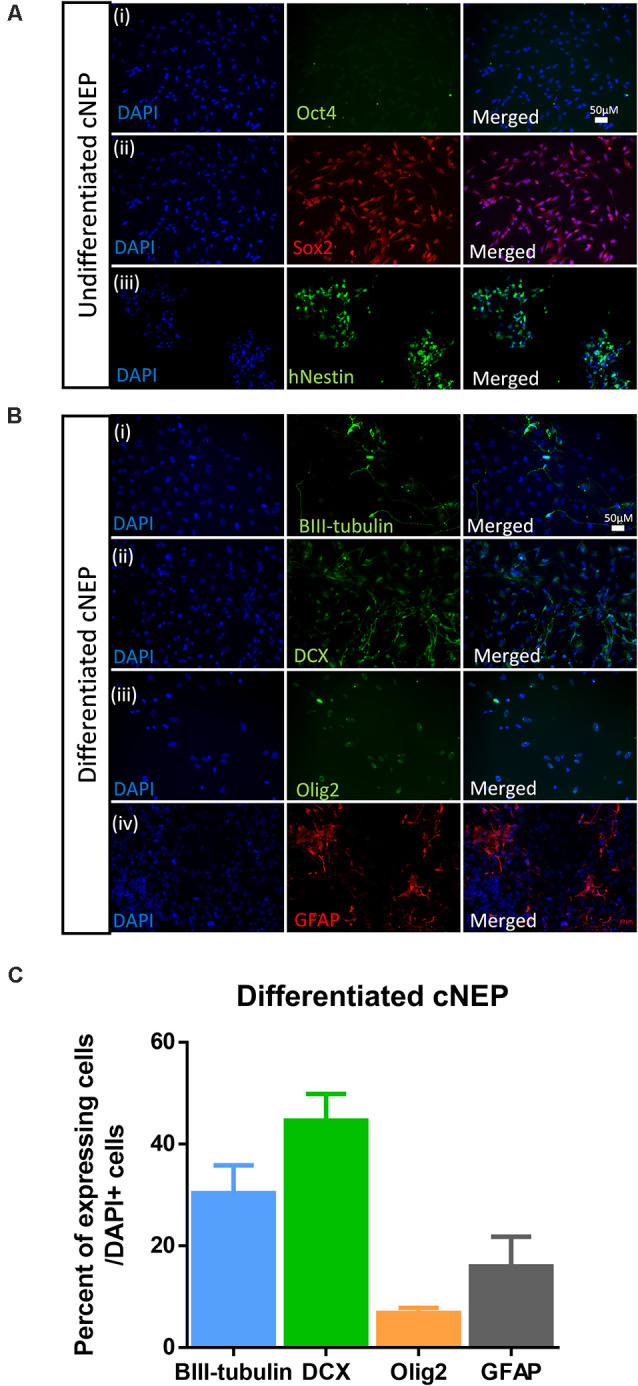
Embryonic stem cell (ESC) derived cortical neuroepithelial cells (cNEPs) are neurally committed, multi-potent cells. **(A)** ESC were induced into cNEPs and then passaged or differentiated in media prior to immunocytochemistry. ESC derived cNEPs do not express the pluripotency marker OCT4 **(i)** and do express the undifferentiated neural precursor markers Sox2 **(ii)** and human Nestin (hNestin) **(iii)**. **(B)** cNEPs were plated on day 0 in differentiation media for 14 days. Subpopulations of cells express BIII-tubulin **(i)**, DCX **(ii)**, Olig2 **(iii)** and GFAP **(iv)**. Scale bars = 50 μm. **(C)** Quantification of differentiation markers. *n* > 3 independent experiments/condition.

ES cells were plated and expanded in ES conditions prior to inducing and plating in cNEP conditions. To examine the neural differentiation potential of the cNEPs plated, cNEPs were collected and replated in maintenance media for 2 days (undifferentiated) or differentiation media for 14 days (differentiated) and immunocytochemistry was performed. Undifferentiated cNEPs expressed the NPC markers Sox2 and Nestin and did not express the pluripotency marker Oct4 expressed by ESCs (Figure 1A). Following 14 days of differentiation, the majority of cNEPs differentiated into DCX + neuroblasts (44.6 ± 5.2%) and BIII-tubulin + immature neurons (30.3 ± 5.5%), with fewer cNEPs expressing the astrocytic marker GFAP (15.9 ± 10.2%) and oligodendryocyte marker Olig2 (6.8 ± 1.7%; Figures 1B,C; Mujtaba et al., 1999; Payne et al., 2019b). Hence, similar to human iPSC derived cNEPs (Payne et al., 2019b), ESC derived cNEPs are multipotent, neurally committed cells *in vitro*.

### Transplanted cNEPs Improve Motor Function Following Stroke

**Figure 2:**
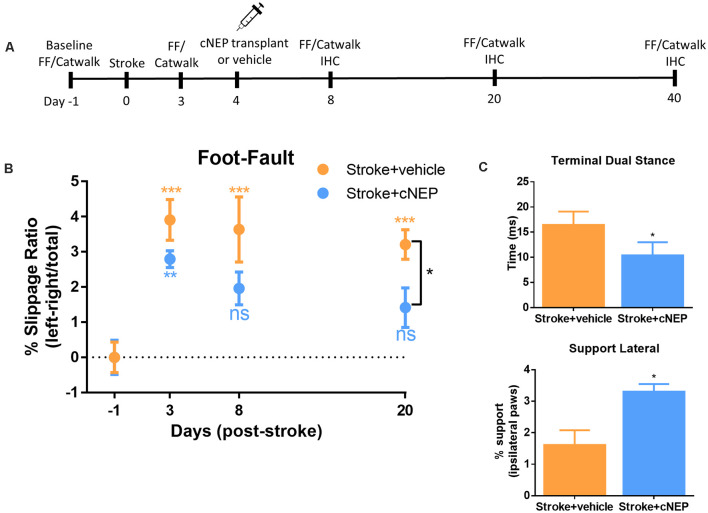
cNEP transplantation improves motor outcomes of stroke by PSD20. **(A)** Schematic of experimental paradigm. FF, foot-fault and IHC, immunohistochemistry. **(B)** The percent slippage ratio was scored in Stroke + vehicle and Stroke + cNEP treated mice. Both groups display a motor deficit on PSD3 and PSD8 compared to baseline performance. Stroke + cNEP mice recover by PSD8 and PSD20 when compared to baseline and is significantly improved compared to Stroke + vehicle mice (Student’s *T*-test, Mann–Whitney *post hoc*). Stroke + vehicle mice maintain the stroke motor deficit on PSD8 and PSD20, compared to baseline. Stroke + cNEP (*n* = 10 mice); Stroke + vehicle (*n* = 10 mice) Two-way ANOVA, Tukey’s *post hoc*. **(C)** Catwalk was used to measure gait parameters after stroke with or without cNEP transplantation. Terminal dual stance measures the duration (ms) of simultaneous contact of both hindpaws. The duration of the left hindlimb terminal dual stance was significantly increased in Stroke + vehicle mice compared to Stroke + cNEP mice. Support lateral is a measurement of the time when ipsilateral paws (lateral forelimb and hindlimb) make simultaneous contact during the walking task Stroke + vehicle mice exhibited significantly impaired support lateral gait pattern compared to Stroke + cNEP treated mice (*n*=6 mice/group). All statistics were performed with Two-way ANOVA, Tukey’s *post hoc*. Data shown as mean ± SEM. ns, not significant; *p < 0.05, **p < 0.01, ***p < 0.001.

CNEPs were transplanted into the stroke lesion of immunocompromised SCID-Beige mice on PSD4 (acute phase) following an ET-1 induced stroke in the motor cortex (Vonderwalde et al., 2019). All mice were tested in the foot-fault task prior to stroke to establish baseline behavior, and again on PSD3 prior to cell transplantation (Figures 2A,B). Stroke injured mice displayed motor deficits in foot-fault at PSD3, relative to baseline performance (Figure 2B). On PSD4, one cohort of mice (Stroke + cNEP) received 1 × 10^5^ cNEP cells into the stroke injury site. Stroke only mice received vehicle injections. Motor behavior was assessed in the Stroke + vehicle and Stroke + cNEP groups on PSD8, PSD20 and PSD40 (4, 16 and 36 days post-transplant). As shown in Figure 2B, mice that received cNEPs displayed significantly improved motor outcomes on PSD20 compared to non-transplanted mice and their motor performance was not significantly different from baseline (pre-stroke) values on PSD8 or PSD20. Mice that received cNEP transplant also had improved motor function compared to stroke untreated mice on PSD20 (Figure 2B). Mice that did not receive cNEP transplants continued to show significant motor deficits compared to their baseline on PSD20 (Figure 2B). Notably, the non-transplanted mice underwent spontaneous recovery by PSD40 and their motor function was not significantly different from the Stroke + cNEP group (*p* = 0.93). Hence, cNEP transplantation improved motor function following ET-1 induced motor cortical stroke as early as PSD20.

To assess motor coordination, we used the Catwalk for gait analysis on PSD20. A number of parameters have been shown to be affected following stroke, including terminal dual stance which measure the time that both hindlimbs remain on the platform (Liu et al., 2013; Caballero-Garrido et al., 2017). Similar to previous reports using MCAO stroke, ET-1 stroked mice demonstrated a significant increase in left hindlimb stance on PSD20, that was reduced in mice that received cNEP transplantation (Figure 2C). Support lateral measures the percent of time during the walk that mice are simultaneously using ipsilateral paws (lateral forelimb and hindlimb). Again, similar to what is observed in MCAO stroked mice, ET-1 stroked mice demonstrated a decrease in the percent of the run that hindlimbs and forelimbs simultaneous make contact during locomotion. This parameter also improved with cNEP transplant on PSD20 (Figure 2C). These findings demonstrate that post-stroke impairments in motor coordination as measured by gait analysis are improved following cNEP transplantation. Immature cNEPs rescue motor deficit following a stroke.

**Figure 3:**
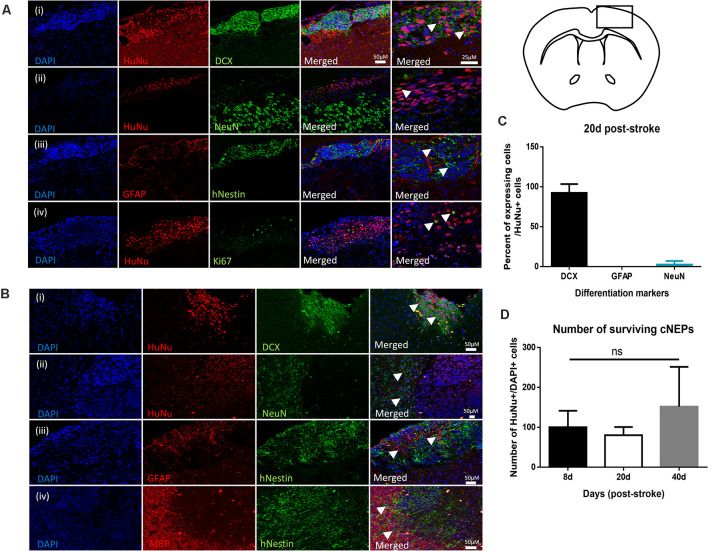
Transplanted CNEPs express doublecortin, a marker of immature neurons. **(A)** Coronal schematic of forebrain at PSD20 HuNu+ or hNestin+ transplanted cNEPs express DCX **(i)**, low levels of NeuN **(ii)** and are GFAP negative **(iii)**. A small number of cells express Ki67 **(iv)**. Scale bar = 25 μm. **(B)** By PSD40, HuNu+ or hNestin+ transplanted cNEPs express DCX **(i)**, but do not colocalize with mature cell markers NeuN **(ii)**, GFAP **(iii)** or MBP **(iv)**. Scale bars = 50 μm. **(C)** Quantification of the differentiation phenotype of HuNu+ cNEP cells in the PSD20 brain showing majority of transplanted cells are DCX + immature neurons. (*n* = 3 mice). **(D)** The total numbers of HuNu+/DAPI+ cells showed no significant difference between the numbers of surviving cells on PSD8 (*n* = 3 mice), PSD20 (*n* = 3 mice) or PSD40 (*n* = 9 mice). Data represents mean ± SEM. One-way ANOVA, Tukey’s *post hoc*; ns, not significant. Arrowheads indicate cells expressing mature or immature neuronal markers.

Given the improved functional outcomes in transplanted mice, we next examined the cellular response in Stroke + cNEP treated mice. We assessed the differentiation profile of the surviving transplanted cells at the time of functional recovery by looking for co-labeled cells expressing human antigen markers, HuNu+ or hNestin+, with markers for mature neurons (NeuN), astrocytes (GFAP) and oligodendrocytes (myelin basic protein (MBP). As shown in Figure 3A, on PSD20, the vast majority of HuNu+ cNEPs expressed the neuroblast marker DCX (92 ± 11%; Figures 3Ai–C), with rare cells expressing the mature neuronal marker NeuN (2.6 ± 4.5%; Figures 3Aii–C). Virtually no HuNu+ cells expressed the astrocyte marker GFAP (Figures 3Aiii–C). We used Ki67 to look for proliferating cells and observed a small number of Ki67+/HuNu+ cNEPs (13 ± 13%) on PSD20 (Figure 3Aiv). As predicted, the contralateral hemisphere of Stroke + cNEP and Stroke only brains did not contain HuNU+ or Ki67+ cells (Supplementary Figure 1). Hence, on PSD20, surviving cNEP derived cells were DCX+, immature neurons.

Since transplanted human cells take longer to differentiate into mature phenotypes, we predicted that mature neurons may develop with longer survival times. We examined the brains of Stroke + cNEP mice on PSD40. Even at this longer survival time, transplanted cells continued to express DCX with rare HuNu+ or hNestin+ cells expressing the mature cell markers NeuN, GFAP or MBP (Figures 3Bi–iii). The contralateral hemisphere of Stroke + cNEP and Stroke only brains did not contain transplanted or proliferating cells (Supplementary Figure 2).

The number of surviving transplanted cells was quantified on PSD8 (a time when functional deficits were observed), PSD20 and PSD40 (when functional recovery is observed). The percentage of surviving cells (HuNu+ and DAPI+) relative to the numbers of transplanted cells (100,000 cells/brain) was not significantly different at any time examined (Figure 3D). Most striking, only three of the nine mice examined on PSD40 had surviving cNEPs (HuNu+ or hNestin+ cells). Hence, while Stroke + cNEP mice displayed improved motor function after PSD20, the recovery was not coincident with surviving cells on PSD40.

### CNEP Transplantation Does Not Affect Stroke Lesion Volume or the Microglia/Macrophage Response

**Figure 4:**
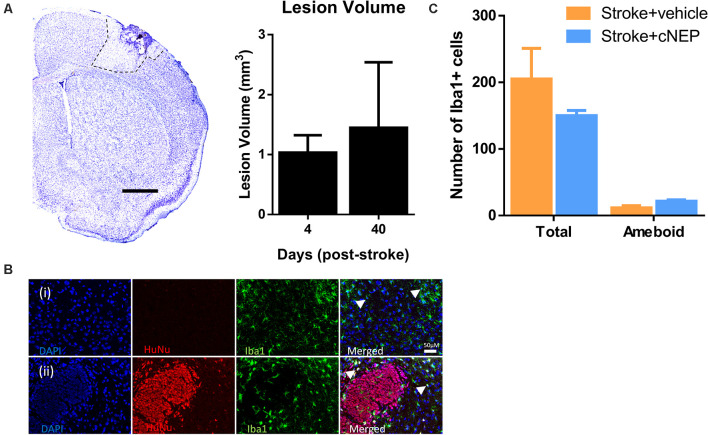
Transplanted cells do not alter the lesion volume or the inflammatory cell response. **(A)** Cresyl violet cross section of the stroke injured cortex on post-stroke day 4 (PSD4). Dotted line delineates the stroke lesion. The lesion volume is not significantly different between PSD4 (pre-transplant) and PSD40 (Stroke + cNEPs). *n* = 3 mice/group; data represents mean ± SEM; Student’s *t*-test. Scale bar = 500 μm. **(B)** Iba1+ inflammatory microglia were stained in Stroke+ vehicle **(i)** and Stroke + cNEP **(ii)** treated mice on PSD8. Scale bar = 50 μm. **(C)** Quantification of iba1+ cells showed no significant difference in the number of Iba1+ cells or activated Iba1+ cells between groups (*n* = 3 mice/group). Data represents mean ± SEM; Student’s *t*-test. Arrowheads indicate iba1+ amoeboid shaped cells.

We next asked if the lesion volume was affected by the transplantation and associated with functional improvement. Cresyl violet stained images were quantified from cNEP transplanted mice sacrificed on PSD4 (prior to transplant) and PSD40. Cortical lesion volumes were not different between PSD4 and PSD40 in Stroke + cNEP transplanted mice (Figure 4A). Hence, cell transplantation does not lead to a change in the lesion volume at a time of functional recovery.

To determine whether cNEP transplantation affected the immunological response in stroke injured mice, the number of Iba1+ inflammatory cells (microglial and macrophages) were counted in the lesional and perilesional stroke injured cortex. SCID-beige mice lack the adaptive immune response but retain the innate immune response (Figure 4B). We observed no difference in the numbers of Iba1+ cells or the number of activated ameboid Iba1+ cells in Stroke only and Stroke + cNEP transplanted mice on PSD8, a time when immune cell activity is robust in response to stem cell transplantation (Walczak et al., 2007; Figures 4B,C). Hence, cNEP transplantation post-stroke does not elicit an enhanced neuroinflammatory response compared to Stroke alone.

## Discussion

Stem cell transplantation is a promising approach to treat the stroke injured brain. Despite the promise, a number of challenges still exist including the identification of an appropriate cell source, the location and timing of transplantation, and a clear understanding of the mechanisms that underlie the potential efficacy of the approach (Bliss et al., 2007). Here, we demonstrate that the acute transplantation of cNEPs in an ET-1 motor cortex stroke model can promote functional recovery as early as 16 days post-transplantation. We found that only a small fraction of the transplanted cells survive in the stroke injury site at the time of recovery, and despite their ability to differentiate into mature phenotypes *in vitro*, the surviving cNEPs remained immature *in vivo*, at the time of functional recovery. Further, we demonstrate that improved functional outcomes are not dependent on the persistence of the transplanted cells once improved functional outcomes have been achieved.

Cell integration and replacement are recognized as mechanisms for functional recovery (Englund et al., 2002; Falkner et al., 2016; Palma-Tortosa et al., 2020). This mechanism requires long term survival, maturation, and integration of transplanted cells to form synaptic connectivity with endogenous cells for functional benefits. Our study demonstrates that cNEP survival, maturation and integration are not required for the by-stander effect observed at early timepoints that give rise to the recovery we observed in this ET-1 stroke model. This is consistent with previous studies using human directly reprogrammed neural precursor cells in the same ET-1 mouse model of stroke (Vonderwalde et al., 2019). The complete absence of cells on PSD40 in 67% of the recovered mice, in combination with the lack of mature neurons suggests that cNEPs support recovery through “by-stander” effects such as secreting factors that enhance host neuroplasticity and cell maturation is not needed to maintain behavioral recovery (Xiong et al., 2017). The release of neurotrophic factors could significantly impact the stroke niche to induce angiogenesis, decrease immunogenic response and improve endogenous stem cell response (Horie et al., 2011; Oliveira et al., 2016). Indeed, the well documented ability of mesenchymal stem cell transplantation to support brain repair post-stroke is shown to be mediated by enhancing brain plasticity and/or reducing the inflammatory response (Kurozumi et al., 2005; Noh et al., 2016; Forsberg et al., 2020; Venkat et al., 2020). Identifying the factors that allow for recovery will be beneficial in determining future avenues of stroke treatment.

The differentiation profile of transplanted cNEPs suggests that unlike previous transplant studies, cNEPs do not contribute to the glial scar by turning into GFAP+ astrocytes (Payne et al., 2019b; Vonderwalde et al., 2019). Astrocytes have been shown to be beneficial following brain injury by limiting the extent of damage, releasing neurotrophic factors and clearing excess glutamate (Barreto et al., 2012; Liu and Chopp, 2016). However, astrocytes are also capable of releasing proinflammatory factors and inhibitory factors, such as chondroitin sulfates, that impair axonal growth (Barreto et al., 2012). Once activated, astrocytes exist within the brain in either anti-inflammatory or pro-inflammatory states (Giovannoni and Quintana, 2020) and reducing pro-inflammatory astrocyte activation has been shown to be beneficial for both behavioral and cellular outcomes after injury (Brambilla et al., 2005). Since the nature of the astrocytes derived from transplanted cells is not known, it is possible that the lack of CNEPs derived astrogliosis is advantageous.

Our study found that a small number of cNEPs expressed the proliferation marker Ki67, which is consistent with the previous study transplanting cNEPS into stroke injured rats (Payne et al., 2019b). Despite the positive functional outcomes and the fact that majority of mice had no surviving cells on PSD40, the presence of proliferating cells can be a concern for clinical application. Ablating proliferating cells using gene editing technologies that result in cell death upon expression of a cell division gene, CDK1, have been recently described (Liang et al., 2018; Payne et al., 2019a) and incorporating this Failsafe system into cNEPs would undoubtedly enhance their clinical application.

CNEP’s can be derived from pluripotent stem cells, including iPSCs (Payne et al., 2018, 2019b) and ES cells (reported here). A previous report using iPSC derived cNEPs embedded in a hydrogel and transplanted in a subacute rat model of motor stroke, demonstrated limited improvements in functional recovery at >50 days post-stroke (Payne et al., 2019b). Payne et al. (2019b) found that most of the cNEPs survive following transplantation (on PSD50) and the majority differentiated into astrocytes. Several differences between the studies are noted, including the cell origin of cNEPs. IPSCs and ESCs have been shown to generate different progeny in the same culture conditions (Kim et al., 2010). Additionally, in the rat study cNEPs were delivered in a hydrogel which would modify the interaction of cNEPs with the host environment, potentially impacting the time of recovery and the differentiation profile of the transplanted cells. Further, the rat stroke model included cortical and subcortical ET-1 lesions, with the cell transplantation being limited to the cortex. Given that cNEPs are cortically specified, the limited recovery observed in their study may be related to the fact that striatal circuitry was disrupted and not impacted by the cNEP transplant. Together, the outcomes of the *in vivo* studies comparing cells that behave similarly *in vitro*, highlight the importance of following the recommendations of STEPS and STAIRS that underscore the need to compare outcomes across different models, with a view towards translation (Lapchak et al., 2013).

When considering stem cell transplantation studies to treat stroke, there are a number of design parameters that can influence outcomes and make it challenging to compare across studies. For example; the cell source, the numbers of cells transplanted, the location and time of transplantation post-stroke and the vehicle used to transplant cells. Our study paradigm was based on the findings that stem cell transplantation into the stroke lesion provided greater survival and functional improvement following stroke, when compared to transplantation into surrounding parenchyma following ET-1 stroke (Ballios et al., 2015). In addition, we have found that the use of a hydrogel (HAMC) did not provide improved survival of human neural stem cells when transplanted into SCID-beige immunodeficient mice (Vonderwalde et al., 2019). Building on these studies we transplanted human cNEPs into the stroke lesion site suspended in regular cerebrospinal fluid in SCID-beige immunocompromised mice. Importantly, this method allowed us to avoid pharmacological immunosuppression, as these treatments have been shown to regulate endogenous neural precursor behavior which could influence the outcomes (Sachewsky et al., 2014). These experimental considerations are important for ensuring best approaches in pre-clinical stroke research.

Our study demonstrates that cortically specified, neurally committed neural precursor cells can promote rapid functional recovery when delivered in the acute phase post stroke without integration into the host circuitry. Future studies will determine the critical window of repair and identify the mechanism that serves to enhance neuroplasticity and functional recovery.

## Data Availability Statement

The raw data supporting the conclusions of this article will be made available by the authors, without undue reservation.

## Ethics Statement

The animal study was reviewed and approved by University of Toronto, Temerty Faculty of Medicine Animal Care Committee.

## Author Contributions

RI designed and performed the experiments, data analysis, interpretation, wrote and edited the manuscript. BV and AN developed the cell culturing protocol. SD and IV performed experiments and data analysis. CM designed experiments and interpreted data, provided financial support, wrote and edited the manuscript. All authors contributed to the article and approved the submitted version.

## Conflict of Interest

The authors declare that the research was conducted in the absence of any commercial or financial relationships that could be construed as a potential conflict of interest.
