# Supplementary material for: Transplantation of Human Cortically-Specified Neuroepithelial Progenitor Cells Leads to Improved Functional Outcomes in a Mouse Model of Stroke
Source: Front Cell Neurosci. 2021 Apr 29;15:654290. doi: 10.3389/fncel.2021.654290 (PMC8116536; doi:10.3389/fncel.2021.654290)
Supplement: Supplementary file 1 [file Data_Sheet_1.docx]

Supplementary Material


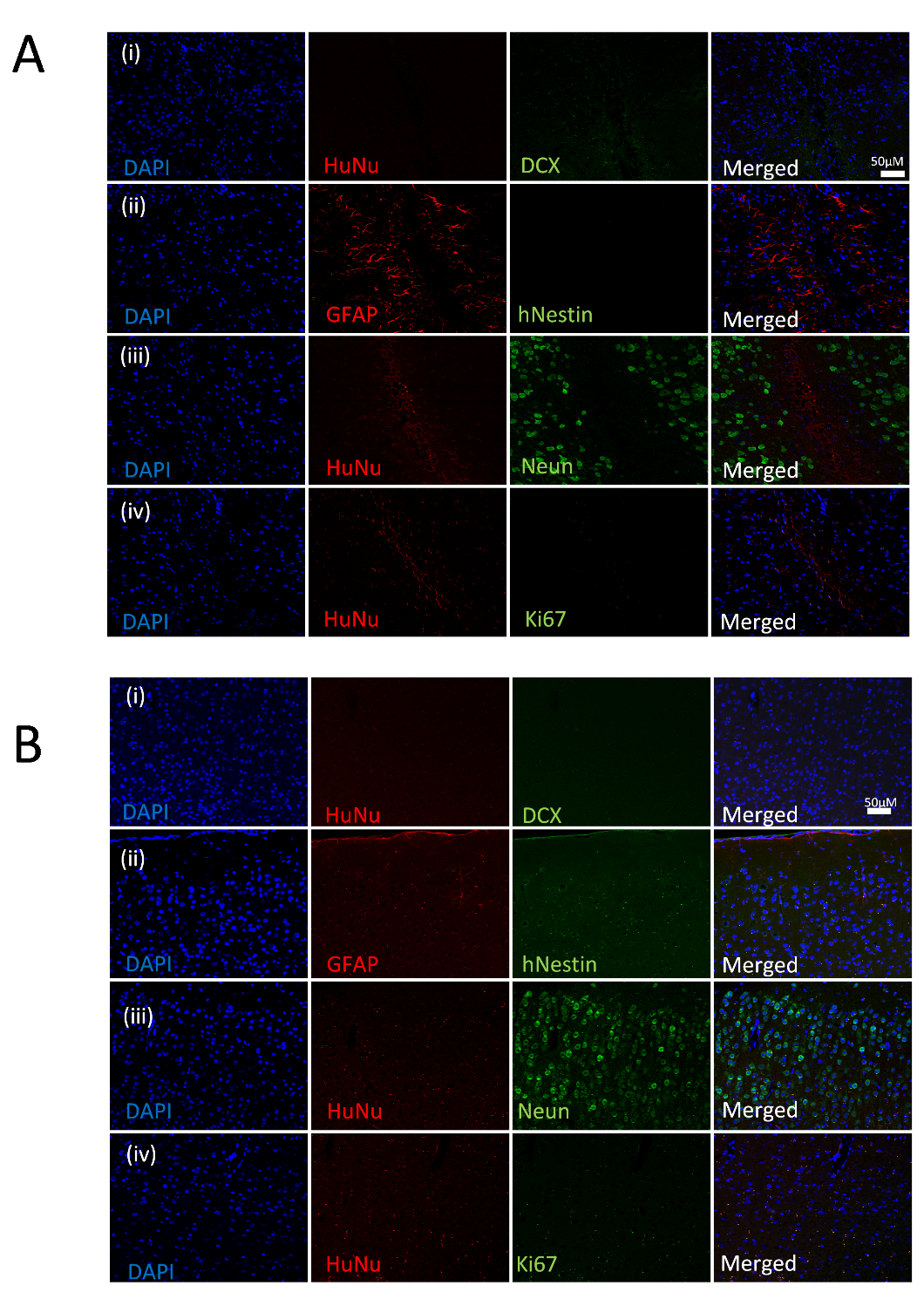


**Supplementary Figure 1.** **The ipsilateral hemisphere of stroke only mice and the contralateral hemisphere of stroke+cNEP mice do not express human antigen markers at PSD20.** The same panel of markers used for in vivo immunohistochemistry was applied to Stroke only tissue and expression was examined in the contralateral Stroke + cNEP hemispheres. **(A)** Stroke only mice do not express human antigen markers HuNu (i) and hNestin (ii). No DCX (i) and Ki67 (iv) expression was observed at PSD20. Stroked mice displayed GFAP+ (ii) astrocytes around the stroke lesion and a loss of Neun+ cells at the lesion site (iii). Scale bar = 50µm. **(B)** The contralateral hemisphere of Stroke + cNEP mice do not express HuNu (i), DCX (i), GFAP (ii), hNestin (ii) or Ki67 (iv). Neun+ (iii) cells are observed in the contralateral hemisphere. Scale bar = 50µm.


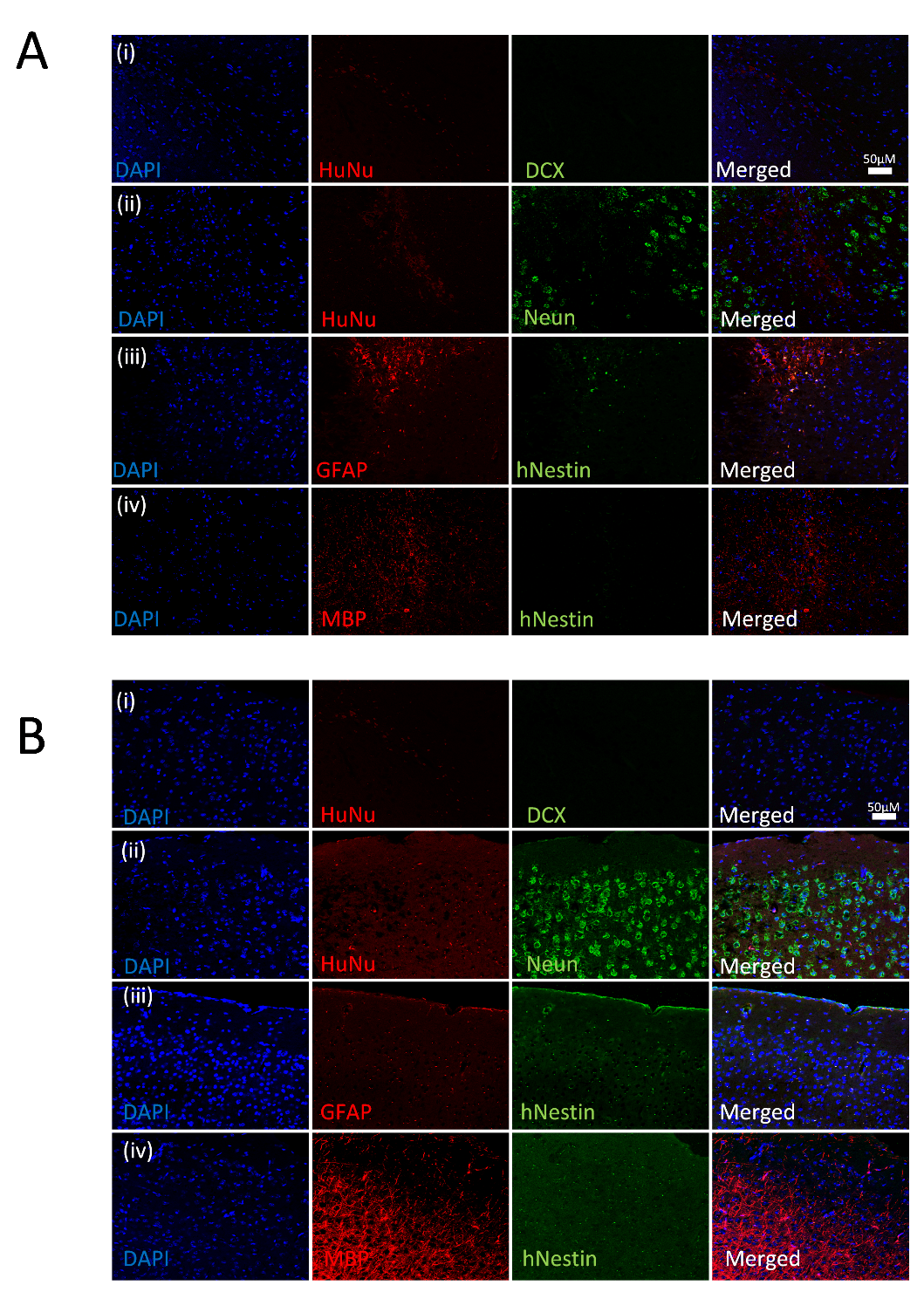


**Supplementary Figure 2.** **The ipsilateral hemisphere of stroke only mice and the contralateral hemisphere of stroke+cNEP mice do not express human antigen markers at PSD40.** The same panel of markers used for in vivo immunohistochemistry was applied to Stroke only tissue and expression was examined in the contralateral Stroke + cNEP hemispheres. **(A)** Stroke only mice do not express humn antigen markers HuNu (i, ii) and hNestin (iii, iv). No DCX (i) is observed at PSD40. Neun+ (ii) cells were lost and GFAP+ (iii) astrogliosis occurred in the stroke injured cortex. MBP (iv) is expressed throughout the brain. **(B)** The contralateral hemisphere of Stroke + cNEP treated mice do not express HuNu (i, ii) or hNestin (iii, iv). Scale bars = 50µm.
